# Solitary cystic metastatic lymph node of occult human papillomavirus-related oropharyngeal cancer mimicking second branchial cleft cyst: A case report

**DOI:** 10.1097/MD.0000000000017800

**Published:** 2019-11-01

**Authors:** Han-Hsuan Liang, Chia-Yuen Chen, Wei-Yu Chen, Tsung-Ming Chen, Wing P. Chan

**Affiliations:** a Department of Radiology, Wan Fang Hospital; b Department of Radiology, School of Medicine, College of Medicine, Taipei Medical University; c Department of Pathology, Wan Fang Hospital; d Department of Pathology, School of Medicine, College of Medicine, Taipei Medical University, Taipei; e Department of Otolaryngology, Shuang Ho Hospital, New Taipei City, Taiwan.

**Keywords:** cystic cervical lymph node metastasis, human papillomavirus-related oropharyngeal cancer, occult primary, second branchial cleft cyst, septation

## Abstract

**Rationale:**

Human papillomavirus (HPV)-related oropharyngeal cancer is becoming more common, the primary cancer AQ4 usually occult and appearing only as cystic cervical lymph node (LN) metastasis. Distinguishing between a benign cystic lesion and cystic LN metastasis is challenging given their similar radiologic and histologic appearances.

**Patient concerns:**

A 54-year-old man presented with a bulging cystic mass measuring 6.4cm on the right side of neck.

**Diagnoses:**

Postexcision diagnosis was second branchial cleft cyst. After 2 years, the cystic mass recurred, and HPV-related tonsillar squamous cell carcinoma with cystic metastatic LNs was confirmed after wide tonsillectomy and neck dissection. The previous cystic lesion proved to be a cystic metastatic LN from the same malignancy with additional p16 immunostain.

**Interventions:**

The patient was treated with adjuvant concurrent chemoradiation therapy.

**Outcomes:**

The patient was followed up in the outpatient department with no evidence of recurrence after 1 year.

**Lessons:**

When an adult has a cystic mass in the upper neck, we must rigorously exclude it as a cystic metastatic LN of occult HPV-related oropharyngeal cancer. Additional p16 staining might be helpful.

## 1 Introduction

Human papillomavirus (HPV)-related oropharyngeal cancer is a distinct disease that has increased in incidence over recent decades. In a meta-analysis, its incidence was shown to rise from 40.5% before 2000 to 64.3% between 2000 and 2004, to 72.2% after 2005 in Europe and North America.^[1]^ It is a sexually transmitted virus, and the genotype of HPV16 oral infection is responsible for carcinogenesis of the oropharynx.^[2]^ On imaging, HPV-related oropharyngeal cancer could be occult in the primary malignancy and appear as only cystic cervical lymph node (LN) metastases. In a single-institution retrospective study,^[3]^ up to 90% of metastatic LNs in squamous cell carcinomas (SCCs) with unknown primary tumors were HPV-positive. In most patients, the primary tumors were eventually found in the oropharynx. The cystic metastatic LNs are usually round or ovoid, homogeneous, and well-defined with an enhancing wall. If the cystic metastatic LN is solitary, it can hardly be distinguished from other benign lesions including branchial cleft cyst. Delayed diagnosis of the malignant disease is associated with a poor survival rate.^[4]^ Here, we present a case of occult HPV-related oropharyngeal cancer and solitary cystic LN metastasis with clinical, radiological, and pathological findings that mimic second branchial cleft cyst. To our knowledge, the cystic metastatic LN in our case is the largest cystic lesion reported in the literature.

## 2 Case report

A 54-year-old man visited the *outpatient department* with the chief complaint of a month-old bulging mass in the upper right part of his neck. Neither tenderness nor fever was present. He had an upper respiratory infection about 1 month prior, and he smoked a half-pack of cigarettes daily over the previous 36 years. Physical exam revealed a palpable painless mass at the upper right side of his neck. Neck sonography showed a cystic lesion in that area. Under the initial impression of a benign entity such as branchial cleft cyst, ultrasound-guided aspiration was performed. Ten milliliters of serous fluid was aspirated, and cytology reported macrophages, lymphocytes, a few neutrophils, and eosinophils, suggestive of inflammation. Patient follow-up was recommended.

**Figure 1:**
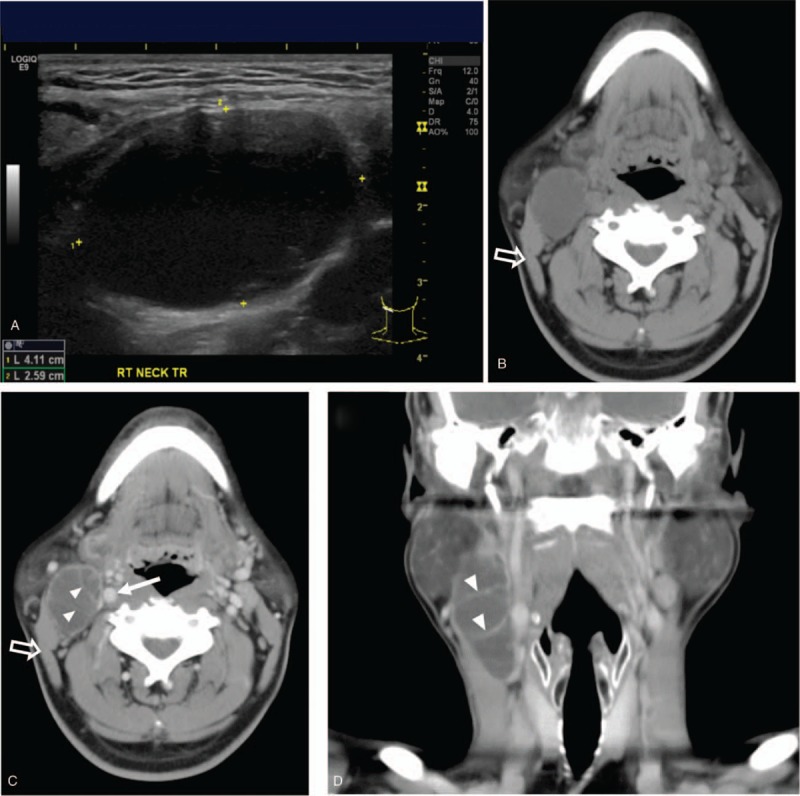
Preoperative image findings. (A) Neck sonography shows a cystic mass in the submandibular region measuring 4.11 cm. (B) Axial non-contrast computed tomography (CT) image, (C and D) axial and coronal contrast-enhanced CT images, respectively, show a cystic mass measuring 6.4 × 4.0 × 2.9 cm with septations (arrowheads) anterior/medial to the sternocleidomastoid muscle (outline arrows) and lateral to the carotid artery (white arrow).

**Figure 2:**
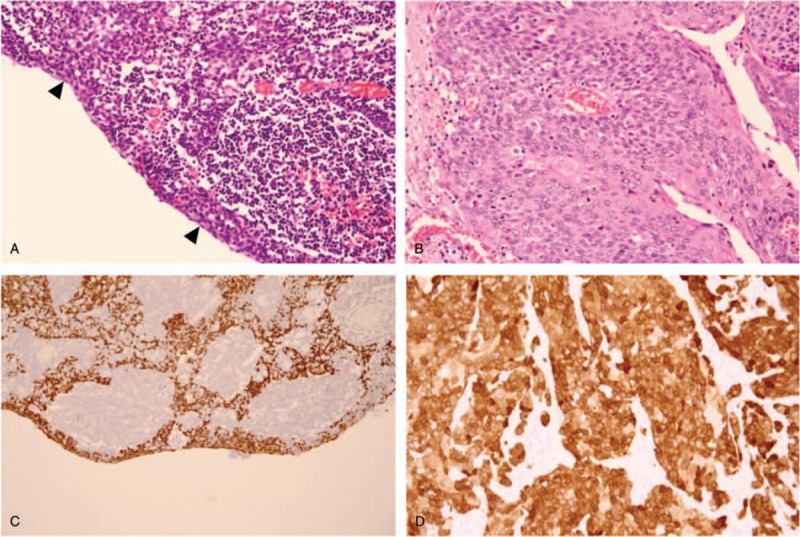
Histological and immunohistochemical findings. (A) On the right side of the neck, a cystic mass lined by a thin stratified squamous epithelium (arrowheads) with minimal nuclear pleomorphism and lymphocyte infiltration in the underlying stroma, morphologically resembling a branchial cleft cyst (hematoxylin and eosin stain; original *magnification*, 100×). (B) Squamous cell carcinoma (SCC) of the right palatine tonsil consisting of tumor cells with mild nuclear atypia (hematoxylin and eosin stain; original *magnification*, 200×). (C) Diffuse strong positive p16 staining in squamous epithelial cells from the cystic mass on the right side of the neck, indicating a metastatic HPV-positive SCC (immunohistochemical stain; original *magnification*, 100×). (D) Diffuse strong positive p16 stain in the right tonsillar SCC (immunohistochemical stain; original *magnification*, 200×).

After 3 months, the mass increased in size. At the second visit, sonography showed a cystic mass in the upper right part of the neck measuring 4.11 cm, comparatively larger than that found during the previous exam (Fig. 1A). The otolaryngologist performed a physical exam and a *fiberscope* exam of the laryngopharynx, and a suspicious mucosal lesion was not found. Head and neck computed tomography showed a cystic mass measuring 6.4 × 4.0 × 2.9 cm with septations anterior/medial to the right *sternocleidomastoid muscle* and lateral to the carotid artery (Fig. 1B–D). Lesions were not noted in the thyroid gland. Under the impression of a benign entity, tumor excision was performed. Microscopic examination revealed a multilocular cyst lined by a stratified squamous epithelium (Fig. 2A). A high nucleus-to-cytoplasm ratio was found, but significant nuclear pleomorphism was not found in the squamous epithelial cells. Lymphocyte infiltration was noticed in the subepithelial stroma. Branchial cleft cyst was diagnosed.

**Figure 3:**
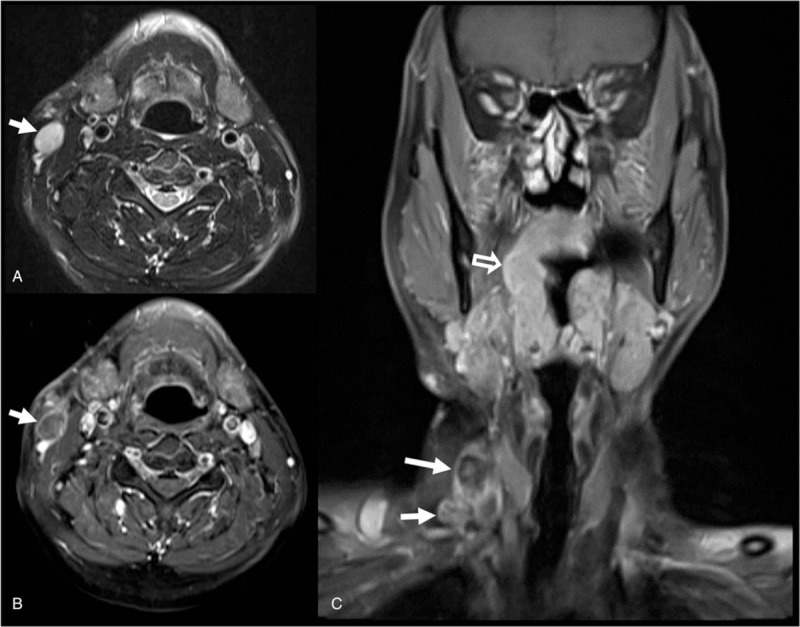
*Follow-up head and neck* magnetic resonance images. (A) Axial T2-weighted image and (B) axial contrast-enhanced T1-weighted image. Both show a recurrent cystic lesion (arrow) measuring 2.19 × 1.82 × 1.35 cm between the platysma and sternocleidomastoid muscle at the hyoid bone level. (C) Coronal contrast-enhanced T1-weighted image with fat suppression shows new cystic lesions at Level IV (arrows) and Level I (not shown). Prominent right palatine tonsil is also noted (outline arrow).

Two years later, the patient returned with a 1-month history of swelling in the upper right part of the neck. Odynophagia was not present. The physical exam revealed the *right palatine tonsil oozed when palpated. Follow-up head and neck* magnetic resonance imaging demonstrated a recurrent cystic lesion at the upper neck near the previous incision site and new cystic lesions on the right side of the neck (at Levels I and IV) with a prominent right palatine tonsil (Fig. 3). Fine needle aspiration cytology of the relapsed cystic mass again showed benign nonspecific cellular change. Under the clinical suspicion of right tonsillar cancer with LN metastasis on the right side of the neck, a wide tonsillectomy was performed, and tonsillar cancer was confirmed by frozen section. A neck dissection at Levels I-IV was subsequently performed on the right side. An ulcerative, firm, and gray-colored tumor measuring 2.8 × 1.6 × 1.4 cm was identified in the tonsil. Microscopically, the tumor showed SCC composed of tumor cells with mild to moderate nuclear pleomorphism (Fig. 2B). The tumor cells were immunoreactive to p16 (Fig. 2D). Human papillomavirus-associated SCC was diagnosed. Metastatic SCC was found in the right nodes at Levels I, II, III, and IV. A retrospective histologic review and p16 immunostain of the previous cystic lesion from the right side of the neck, excised 2 years prior, were performed. A positive p16 stain was seen in the squamous epithelium. The pathologic diagnosis was revised to metastatic SCC (Fig. 2C). The patient was treated with a*djuvant* concurrent chemoradiation therapy and followed-up in the outpatient department with no evidence of recurrence after 1 year. The patient has provided informed consent for the publication of this case report and accompanying images.

## 3 Discussion

To date, HPV-related oropharyngeal cancer is well known for its varying epidemiology, etiology, clinical presentation, and prognosis. Patients commonly present with palpable painless neck masses.^[5]^ Male predilection, younger age (average, 57.3 years),^[3]^ and lack of smoking are characteristics in epidemiology. Recently, HPV-related oropharyngeal SCC has been described as associated with cystic LN metastasis.^[6]^ The primary cancer is small or occult and mostly arises from a tonsil or the base of the tongue. It generally occurs in patients at least 40 years old. However, the cystic LNs in these reports were multiple or single and no larger than 6 cm in size. To our knowledge, this is the first report of a case of a single huge metastatic LN in the neck without soft tissue components or necroses.

Second branchial cleft cyst usually presents at the mandibular angle anterior to the sternocleidomastoid muscle.^[7,8]^ Its cause is incomplete obliteration of the branchial apparatus, resulting cysts, sinuses, and fistulas.^[9]^ It grows slowly and becomes evident in the second or third decade of life. It is painless and mobile. It can become swollen, infected, and inflamed shortly after an upper respiratory tract infection, odontogenic infection, or pregnancy.^[9,10]^ Images typically show an oval or round cystic mass with a thin and smooth non-enhancing wall or an irregular, thick, enhancing wall if complicated with infection.^[11]^ Although it is a congenital cyst, a 70-year-old patient with a second branchial cleft cyst has been reported.^[12]^

In differentiating a benign branchial cleft cyst from malignant cystic metastatic LNs on images, Goyal et al suggested that branchial cleft cysts tend to be larger, homogeneous, with fewer septations, and less extracapsular spread compared to cystic metastatic LNs. In another study, cystic neck masses with internal vascularization, intracystic solid components, and irregular outer walls were suspected to be malignant.^[11]^ As in our case, computed tomography revealed a cystic mass that was homogeneous without extracapsular spread, internal vascularization, intracystic solid components, or irregular outer walls. The size was 6.4 cm, much larger than the largest cystic metastatic LN from Goyal study (2.5 cm). Even though septations were detected, image characteristics primarily resembled a branchial cleft cyst. Septations could be found in 19% of the branchial cleft cysts and 42.1% of the malignant cystic LN.^[13]^ Our case is in agreement with Goyal study, which showed that septations should be viewed as a suspicious feature of malignancy.

Fine needle aspiration is a standard diagnostic tool for a cystic neck mass. However, it reportedly results in false negative results 50% to 67% of the time because hypocellularity in the cyst cavity and inflammatory reactions producing numerous inflammatory cells and cellular debris in the specimen cytologically mimic a branchial cleft cyst.^[14]^ Histologically, a cystic metastatic LN could have a bland appearance without cytological atypia, and the tumor cell could be very thin with only 1 layer of cell lining.^[15]^ This raises the difficulty in differentiating benign lesions from malignancy.^[16]^ Similarly, in our case, cytology presented benign findings twice, and the initial pathology showed a cyst lined with a stratified squamous epithelium without evidence of cell atypia, leading to the presumption of a branchial cleft cyst. After p16 immunohistochemical staining, diffuse positivity was revealed on a stratified squamous epithelium lining, thus changing the diagnosis to a cystic metastatic LN of SCC. Although reports have stated that a branchial cleft cyst can also show positive p16 staining, it is a weak stain and is limited to the epithelium lining. It is not diffuse staining.^[17]^

A solitary cystic lesion in the upper part of the neck could also be a neurogenic tumor, an infectious or inflammatory lymphadenopathy, or a cystic metastatic LN of another malignancy. Four percent of head and neck neurogenic tumors are cystic. Few case reports describe cystic schwannoma in the neck mimicking second branchial cleft cyst, which can present as a painless, slow-growing mass located at the carotid space shown in images to be a well-circumscribed non-enhancing mass with smooth margins.^[18]^ Infectious lymphadenopathy with cystic changes could be caused by a virus, tuberculosis infection, or cat scratch disease. Inflammatory lymphadenopathy with cystic changes constitute Kikuchi disease. Patients with infectious or inflammatory lymphadenopathy usually present with multiple neck masses accompanied by tenderness and fever. Multiple LNs with obscured perinodal fat planes are seen on images. Among these etiologies, tuberculosis tends to produce painless neck masses, and images show LNs with cystic changes and preserved adjacent fat planes. Kikuchi disease typically affects patients aged less than 30 years. Other infectious diseases can occur at any age. Cystic LN metastases of other malignancies typically occur in thyroid cancer. On imaging, it can present as a thick-walled cystic enhancing mass typically located in the middle and lower regions of the jugular chain. Calcifications might also be present. The primary lesion in the thyroid gland can be occult or too small to be detected in the image.^[19]^

## 4 Conclusion

Nowadays, we know that cystic LN metastasis often occurs in patients without obvious clinical or radiographic primary lesions^[15]^ and is strongly associated with HPV-related oropharyngeal cancer.^[6]^ As a result, in patients who present with a single cystic neck mass, particularly when the image shows suspicious features (such as septations in our case), even if the histopathological findings indicate benign lesions, we should vigorously exclude occult HPV-related oropharyngeal cancer with cystic LN metastasis. Additional p16 staining might be helpful.

## Author contributions

**Conceptualization:** Chia-Yuen Chen, Wei-Yu Chen, Tsung-Ming Chen, Wing P. Chan.

**Writing – original draft:** Han-Hsuan Liang.

**Writing – review & editing:** Chia-Yuen Chen, Wei-Yu Chen, Tsung-Ming Chen, Wing P. Chan.

Chia-Yuen Chen orcid: 0000-0002-9654-4912.
